# Establishment and evaluation of a prediction model for acute gastrointestinal injury in patients with prolonged disorder of consciousness

**DOI:** 10.1186/s12876-022-02536-y

**Published:** 2022-10-25

**Authors:** Wenpei Fu, Zhihang Hu, Xiaomei Zhou, Liang Chen, Mei Wang, Yingying Zhu, Yinliang Qi

**Affiliations:** 1 Department of Hyperbaric Oxygen, The Second People’s Hospital of Hefei, Hefei Hospital Affiliated to Anhui Medical University, Hefei, 230011 Anhui China; 2 Department of Intensive Care Unit, The Second People’ S Hospital of Hefei, Hefei Hospital Affiliated to Anhui Medical University, Hefei, 230011 Anhui China

**Keywords:** Prolonged disorder of consciousness, Acute gastrointestinal injury, Model, Prediction

## Abstract

**Objective:**

To establish a prediction model for acute gastrointestinal injury (AGI) in patients with prolonged disorder of consciousness (pDOC) and to evaluate and apply the prediction model.

**Methods:**

The clinical data of 165 patients with pDOC admitted to the hyperbaric oxygen department from January 2021 to December 2021 were retrospectively reviewed, and the patients were divided into an AGI group (*n* = 91) and an N-AGI group (*n* = 74) according to whether AGI occurred. A prediction model was built by fitting multiple independent influencing factors through logistic regression. The receiver operating characteristic (ROC) curve was used to evaluate the predictive value of the model, the Hosmer–Lemeshow (H–L) test was used to evaluate the goodness-of-fit of the model, and the ROC curve and calibration curve were drawn to evaluate the predictive performance. A nomogram was plotted to visualize the prediction model.

**Results:**

According to the multivariate logistic regression analysis results, the prediction model was finally constructed with the CRS-R score, DAO, PCT, ALB, and I-FABP, and a nomogram was generated. The area under the ROC curve (AUC) of the prediction model was 0.931, the sensitivity was 83.5%, and the specificity was 93.2%. The data were divided into 5 groups for the H–L test (χ^2^ = 2.54, *P* = 0.468 > 0.05) and into 10 groups for the H–L test (χ^2^ = 9.98, *P* = 0.267 > 0.05). A calibration curve was drawn based on the test results, indicating that the prediction model has a good goodness-of-fit and good prediction stability.

**Conclusion:**

The prediction model for AGI in pDOC patients constructed in this study can be used in clinical practice and is helpful to predict the occurrence of AGI in pDOC patients.

## Background

With the continuous improvement of neurology, neurosurgery and critical care medicine, an increasing number of patients with severe craniocerebral trauma and stroke can survive the dangerous period and have prolonged disorder of consciousness (pDOC). pDOC refers to a variety of severe brain injuries resulting in loss of consciousness for more than 28 days [1, 2], including vegetative state/unresponsive wakefulness syndrome (VS/UWS) and minimally conscious state (MCS) [3, 4]. According to a survey, there are more than 100,000 new pDOC patients in China every year [5]. Acute gastrointestinal injury (AGI) is often overlooked during the treatment of pDOC patients. AGI was first proposed by the European Society of Critical Care in 2012. It is defined as gastrointestinal dysfunction caused by acute diseases in critically ill patients and is divided into four grades according to their severity [6]. Patients with AGI have different degrees of gastrointestinal dysfunction, which can cause intestinal flora imbalance, bacterial migration and toxin absorption. If they are not treated quickly and effectively, it can lead to systemic inflammatory response syndrome (SIRS). Eventually, multiple organ dysfunction syndrome (MODS) occurs. Especially for pDOC patients who often have low immune function, AGI may endanger the patient's life. Therefore, the early prediction of AGI is of great significance in pDOC patients. At present, there is no clinical report on a relevant early prediction model. Therefore, this study constructed a combined predictor model by using logistic regression and explored its clinical value in predicting the occurrence of AGI in pDOC patients through retrospective analysis.

## Materials and methods

### Clinical data

Patients with pDOC admitted to the Hyperbaric Oxygen Department of Hefei Second People’s Hospital from January 2021 to December 2021 were reviewed. The inclusion criteria were as follows: ① age ≥ 18 years; ② the Coma Recovery Scale-Revised (CRS-R) score at admission was diagnosed as VS/UWS and MCS; and ③ the duration of consciousness disorder was ≥ 28 days. The exclusion criteria were as follows: ① hospitalization time less than 96 h; ② incomplete clinical data related to the study; ③ previous chronic diseases of the digestive tract system; and ④ previous history of abdominal surgery.

This study complied with the relevant requirements of the Declaration of Helsinki of the World Medical Association, and informed consent was obtained from the patient's authorized principal.

### Methods

#### Definition and diagnostic criteria of AGI

According to the AGI diagnosis and grading standards issued by the European Society of Critical Care Medicine in 2012, the AGI grades were defined as follows:

- ① AGI grade I (presence of risk factors for gastrointestinal dysfunction or failure): There is a clear etiology and temporary, partial impairment of gastrointestinal function. For example, nausea, vomiting and bowel sounds disappeared after abdominal surgery, and intestinal motility weakened in the early stage of shock.
- ② AGI grade II (gastrointestinal dysfunction): The gastrointestinal tract does not have complete digestion and absorption functions and cannot meet the body's needs for nutrients and water. Gastrointestinal dysfunction does not affect the patient's general condition. For example, gastroparesis with massive gastric retention or regurgitation, lower gastrointestinal paralysis, diarrhea, intra-abdominal hypertension (IAH) grade I (intra-abdominal pressure IAP 12–15 mmHg), bleeding in stomach contents or stool, and poor food tolerance.
- ③ AGI grade III (gastrointestinal failure): After intervention, gastrointestinal function still cannot be recovered, and the general condition of the patient does not improve. Examples include persistent food intolerance, massive gastric retention, persistent gastrointestinal paralysis, intestinal dilatation, progression of IAH to grade II (intra-abdominal pressure 15–20 mmHg), and decreased intraperitoneal perfusion pressure (APP) (< 60 mmHg).
- ④ AGI grade IV (gastrointestinal failure with distant organ dysfunction): AGI gradually progresses, MODS and shock progressively worsen, and life is at risk at any time. Examples include intestinal avascular necrosis, gastrointestinal bleeding leading to hemorrhagic shock, Ogilvie’s syndrome, and abdominal compartment syndrome (ACS) requiring aggressive decompression.

#### Grouping

The case data were retrospectively analyzed, and the patients were divided into an AGI group and an N-AGI group according to whether AGI occurred during treatment.

#### Observation indicators

We collected and recorded the clinical data of all patients with pDOC during hospitalization, including age, sex, causative disease of pDOC,routine blood tests at the first admission, blood biochemistry, coagulation function, procalcitonin (PCT), C-reactive protein (CRP), serum amyloid A (SAA), diamine oxidase (DAO), and intestinal fatty acid binding protein (IFABP) test results. We also collected and recorded whether the patients had mechanical ventilation, whether they used sedative and analgesic drugs, whether they used vasoactive drugs, whether they used broad-spectrum antibacterial drugs, whether they underwent anticoagulation therapy, whether they had multidrug-resistant bacterial infection, etc. The Sequential Organ Failure Score (SOFA), and the Coma Recovery Scale–Revised (CRS-R) score were calculated on the first day of admission.

### Statistical methods

All extracted data were preprocessed and statistically analyzed using STATA 15.0 software. Continuous variables were tested for normality first, measurement data that conformed to a normal distribution are expressed as the mean ± standard deviation (X ± S), and independent samples t test was used for comparisons between groups. Nonnormally distributed measurement data are expressed as the median (interquartile range) (M (QR)), and the Mann–Whitney U test was used for comparisons between groups. Count data are expressed as frequency (rate), and the chi-square test was used. The possible risk factors for AGI were screened by univariate analysis, and the independent risk factors for AGI in patients with chronic disorder of consciousness were determined by binary logistic regression analysis. Receiver operating characteristic (ROC) curves and calibration curves were drawn to assess the predictive performance of the model. A nomogram was plotted to visualize the prediction model. *P* < 0.05 was considered statistically significant.

## Results

### Baseline data comparison (Table 1)

**Table 1:** Comparison of clinical data between AGI group and N-AGI group in pDOC patients

Group	AGI group (*n* = 91)	N-AGI group (*n* = 74)	χ^2^/t/Z value	*P* value
Number of cases	91	74		
Male (%)	62 (68.13)	44 (59.46)	1.336	0.25
Age (X ± S)	61.58 ± 12.21	58.74 ± 13.48	1.417	0.16
Causative disease of pDOC				
TBI	14 (15.38)	20 (27.03)	3.381	0.07
Intracerebral hemorrhage	52 (57.14)	44 (59.46)	0.09	0.76
Cerebral infarction	11 (12.09)	3 (4.05)	3.392	0.07
Ischemic hypoxic encephalopathy	14 (15.38)	7 (9.46)	1.290	0.26
CRS-R score [M(QL, QU)]	4 (2, 5)	5 (4, 8)	4.277	< 0.01
SOFA score [ M(QL, QU)]	3 (3, 4)	3 (2, 3)	-5.744	< 0.01
Use of broad-spectrum antibiotics (Number of cases, %)	65 (71.43)	42 (56.76)	3.854	0.05
Multidrug-resistant bacterial infection (Number of cases, %)	35 (38.46)	28 (37.84)	0.007	0.94
Mechanical Ventilation (Number of cases, %)	11 (12.09)	5 (6.76)	1.325	0.25
Use of vasoactive drugs (Number of cases, %)	11 (12.09)	0 (0)	9.584	< 0.01
Use of sedatives (Number of cases, %)	35 (38.46)	26 (35.14)	0.194	0.66
Use of analgesics (Number of cases, %)	1 (1.09)	0 (0)	0.818	0.37
WBC (× 10^9^, $\overline{\mathrm{X} }$±S)	8.49 ± 2.96	7.41 ± 2.45	2.557	0.01
Neutrophil percentage (%, X ± S)	69.56 ± 10.49	67.22 ± 9.44	1.492	0.14
Lymphocyte count (× 10^9^, $\overline{\mathrm{X} }$±S)	1.55 ± 0.75	1.62 ± 0.62	-0.623	0.53
Hemoglobin (g/l, $\overline{\mathrm{X} }$±S)	107.22 ± 19.03	112.02 ± 18.19	-1.643	0.10
Platelets (× 10^9^, $\overline{\mathrm{X} }$±S)	260.31 ± 98.44	258.59 ± 92.33	0.115	0.91
CRP, [mg/L, M(QL, QU)]	16.96 (6.58, 46.33)	9.08 (4.77, 22.00)	-2.770	< 0.01
SAA (mg/l, $\overline{\mathrm{X} }$±S)	87.44 ± 108.64	44.20 ± 66.67	3.127	< 0.01
PCT (ng/l, $\overline{\mathrm{X} }$±S)	0.51 ± 0.60	0.14 ± 0.17	5.596	< 0.01
Prothrombin time (s, $\overline{\mathrm{X} }$±S)	13.22 ± 1.21	12.95 ± 0.97	1.522	0.13
Activated partial thromboplastin time (s, $\overline{\mathrm{X} }$±S)	35.94 ± 7.18	35.12 ± 5.46	0.805	0.42
D-dimer (μg/mL, $\overline{\mathrm{X} }$±S)	2.90 ± 4.19	1.53 ± 1.83	2.802	< 0.01
Serum potassium [mmol/L, M (QL, QU)]	4.11 (3.77, 4.37)	3.92 (3.63, 4.22)	-2.312	0.02
ALB [g/L, M(QL, QU)]	33.1 (30.6, 36.6)	35.05 (32.48, 38.68)	2.865	< 0.01
Aspartate aminotransferase (U/L, $\overline{\mathrm{X} }$±S)	40.33 ± 33.18	31.73 ± 28.32	1.796	0.07
Alanine aminotransferase (U/L, $\overline{\mathrm{X} }$±S)	49.48 ± 70.17	35.39 ± 40.04	1.536	0.13
Blood urea nitrogen (mmol/L, $\overline{\mathrm{X} }$±S)	6.90 ± 5.41	4.82 ± 1.97	3.405	< 0.01
Creatinine (μmol/L, $\overline{\mathrm{X} }$±S)	54.57 ± 37.07	45.20 ± 16.00	-2.173	0.03
Total bilirubin (μmol/L, $\overline{\mathrm{X} }$±S)	11.17 ± 5.04	11.43 ± 4.51	-0.349	0.73
Direct bilirubin (μmol/L, $\overline{\mathrm{X} }$±S)	3.91 ± 3.83	3.19 ± 1.40	1.649	0.10
Indirect bilirubin (μmol/L, $\overline{\mathrm{X} }$±S)	7.62 ± 3.73	8.24 ± 3.45	-1.093	0.28
Lactate dehydrogenase (U/L, $\overline{\mathrm{X} }$±S)	236.35 ± 90.24	197.35 ± 57.88	3.360	< 0.01
Triglycerides [mmol/L, M(QL, QU)]	1.47 (1.17, 2.11)	1.52 (1.14, 2.00)	-0.182	0.86
Cholesterol [mmol/L, M(QL, QU)]	4.10 (3.41, 4.70)	4.26 (3.56, 5.18)	1.407	0.16
Prealbumin [mg/L, M(QL, QU)]	191.5 (153.00, 236.90)	200.50 (150.80, 246.03)	0.473	0.64
Anticoagulant therapy (Number of cases, %)	30 (32.97)	22 (29.73)	0.198	0.66
IFABP (μgl/L, $\overline{\mathrm{X} }$±S)	19.11 ± 3.77	17.57 ± 2.45	3.181	< 0.01
DAO (mmol/L, $\overline{\mathrm{X} }$±S)	1.99 ± 0.53	1.43 ± 0.32	8.248	< 0.01

*CRS-R* is the Coma Recovery Scale-Revised, *SOFA* is Sequential Organ Failure Score, *CRP* is C-reactive Protein, *SAA* is Serum Amyloid A, *PCT* is Procalcitonin, *TBI* is Traumatic brain injuries

A total of 165 pDOC patients were included in this study, including 91 in the AGI group and 74 in the N-AGI group. CRS-R score, SOFA, PCT, albumin (ALB), use of vasoactive drugs, white blood cell count(WBC), CRP, SAA, D-dimer, serum potassium, creatinine, blood urea nitrogen, LDH, IFABP, and DAO were significantly different (*P* < 0.05).

### Multivariate analysis

**Fig.1:**
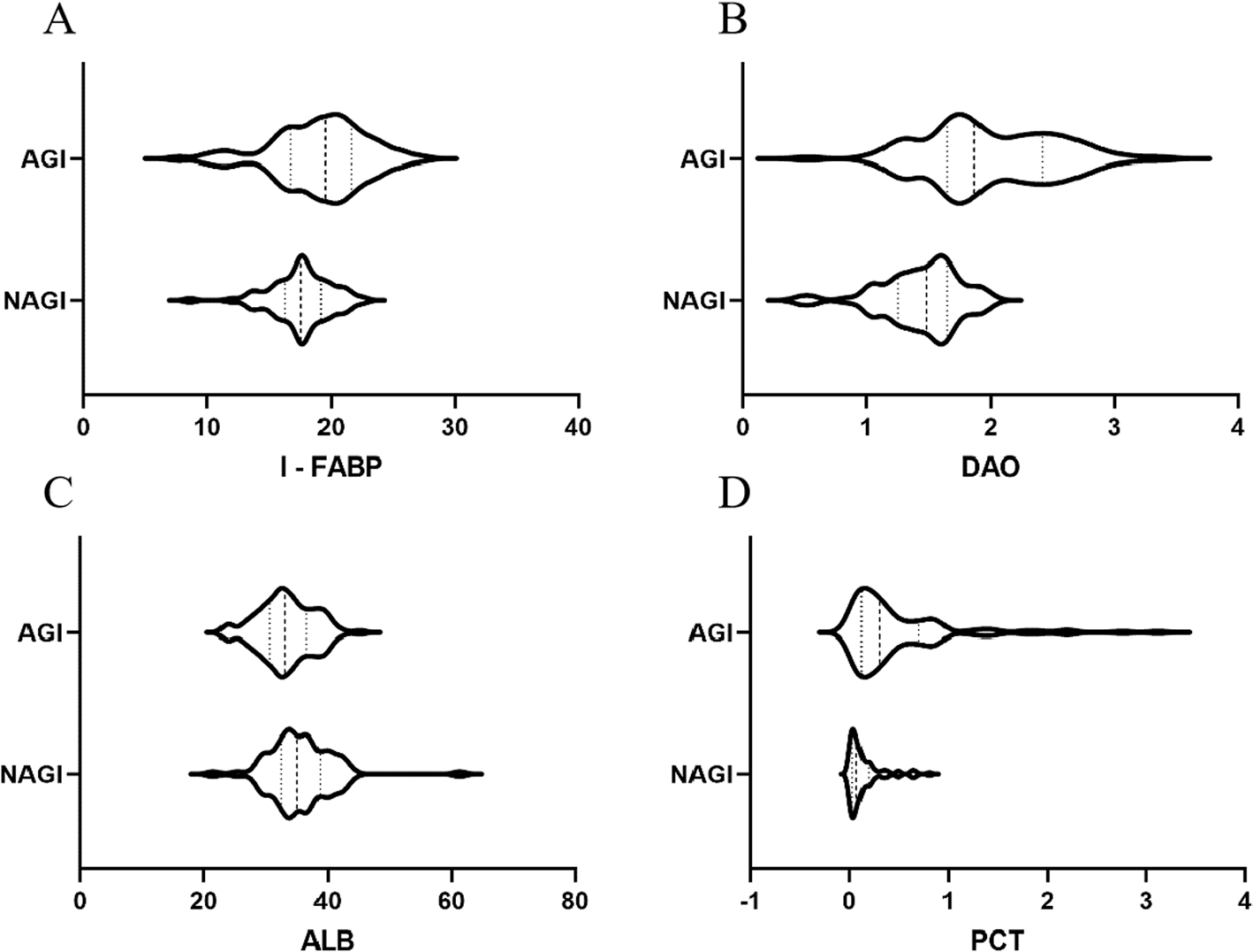
**A** Comparison of I-FABP between AGI group and NAGI group in pDOC patients; **B** Comparison of DAO between AGI group and NAGI group in pDOC patients; **C** Comparison of ALB between AGI group and NAGI group in pDOC patients; **D **Comparison of PCT between AGI group and NAGI group in pDOC patients. I-FABP: intestinal fatty acid binding protein(μgl/L); DAO: diamine oxidase (mmol/L); PCT: procalcitonin(ng/L); ALB: albumin (g/L)

The results of multivariate analysis showed that CRS-R score, DAO, PCT, ALB, and I-FABP were independent influencing factors of AGI in pDOC patients (*P* < 0.05), as shown in Table 2. The IFABP of the AGI group was 19.11 ± 3.77, and the IFABP of the N-AGI group was 17.57 ± 2.45. The DAO of the AGI group was 1.99 ± 0.53, and the DAO of the N-AGI group was 1.43 ± 0.32. The PCT of the AGI group was 0.51 ± 0.60, and the PCT of the N-AGI group was 0.14 ± 0.17. The ALB of the AGI group was 33.35 ± 4.42, and the ALB of the N-AGI group was 35.59 ± 5.31 (Fig. 1). Its effects are as follows: ① For every 1-point increase in the CRS-R score, the probability of AGI in pDOC patients decreases by 0.266; ② For every 1 mmol/L increase in DAO, the possibility of AGI increases by 3.561 times; ③ For every 1 ng/mL increase in PCT, the possibility of AGI increases by 5.144 times; ④ For every 1 g/L increase in ALB, the possibility of AGI decreases by 0.149; ⑤For every 1 μgl/L increase in I-FABP, the possibility of AGI increases by 0.223.

**Table 2:** Multivariate analysis of risk factors to AGI

Factor	OR	β	S.E	P	95%CI
CRSR score	0.766	-0.266	0.088	0.002	-0.438 ~ -0.094
DAO	35.190	3.561	2.564	< 0.001	2.187 ~ 4.935
PCT	171.441	5.144	1.475	< 0.001	2.253 ~ 8.035
ALB	0.862	-0.149	0.056	0.008	-0.258 ~ -0.039
I-FABP	1.250	0.223	0.082	0.006	0.064 ~ 0.383

### Prediction model construction

**Fig. 2:**
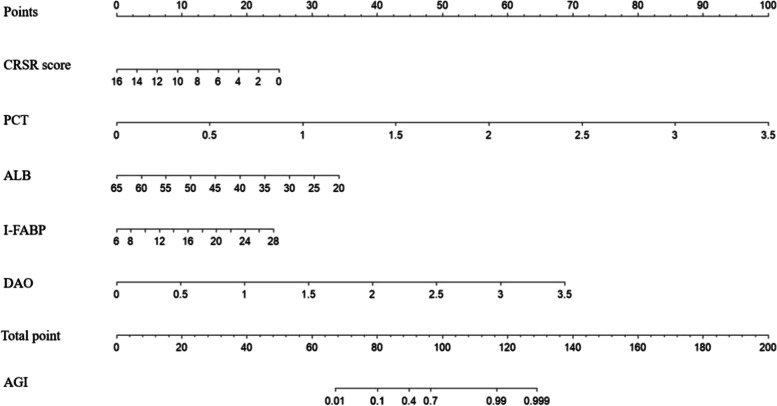
The nomogram for predicting AGI in patients with pDOC. PCT: procalcitonin(ng/L); ALB: albumin (g/L); I-FABP: intestinal fatty acid binding protein(μgl/L); DAO: diamine oxidase (mmol/L)

**Fig. 3:**
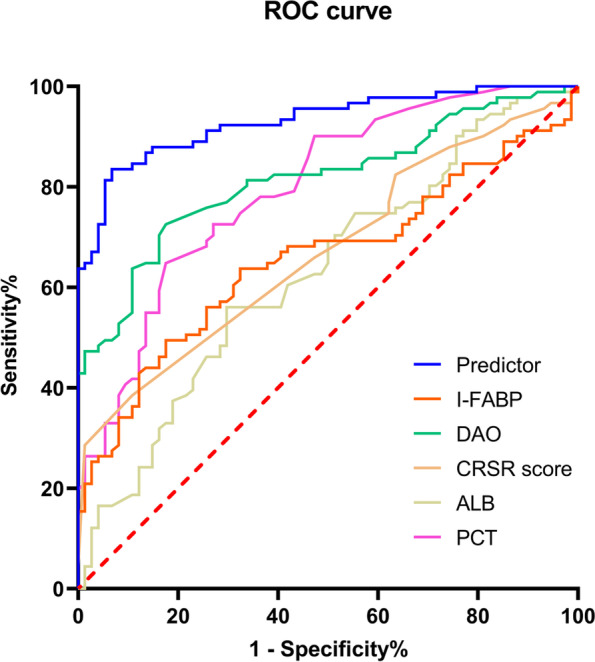
The ROC curve of ROC curves of predictive model for AGI in patients with pDOC. PCT: procalcitonin(ng/L); ALB: albumin (g/L); I-FABP: intestinal fatty acid binding protein(μgl/L); DAO: diamine oxidase (mmol/L)

**Fig.4:**
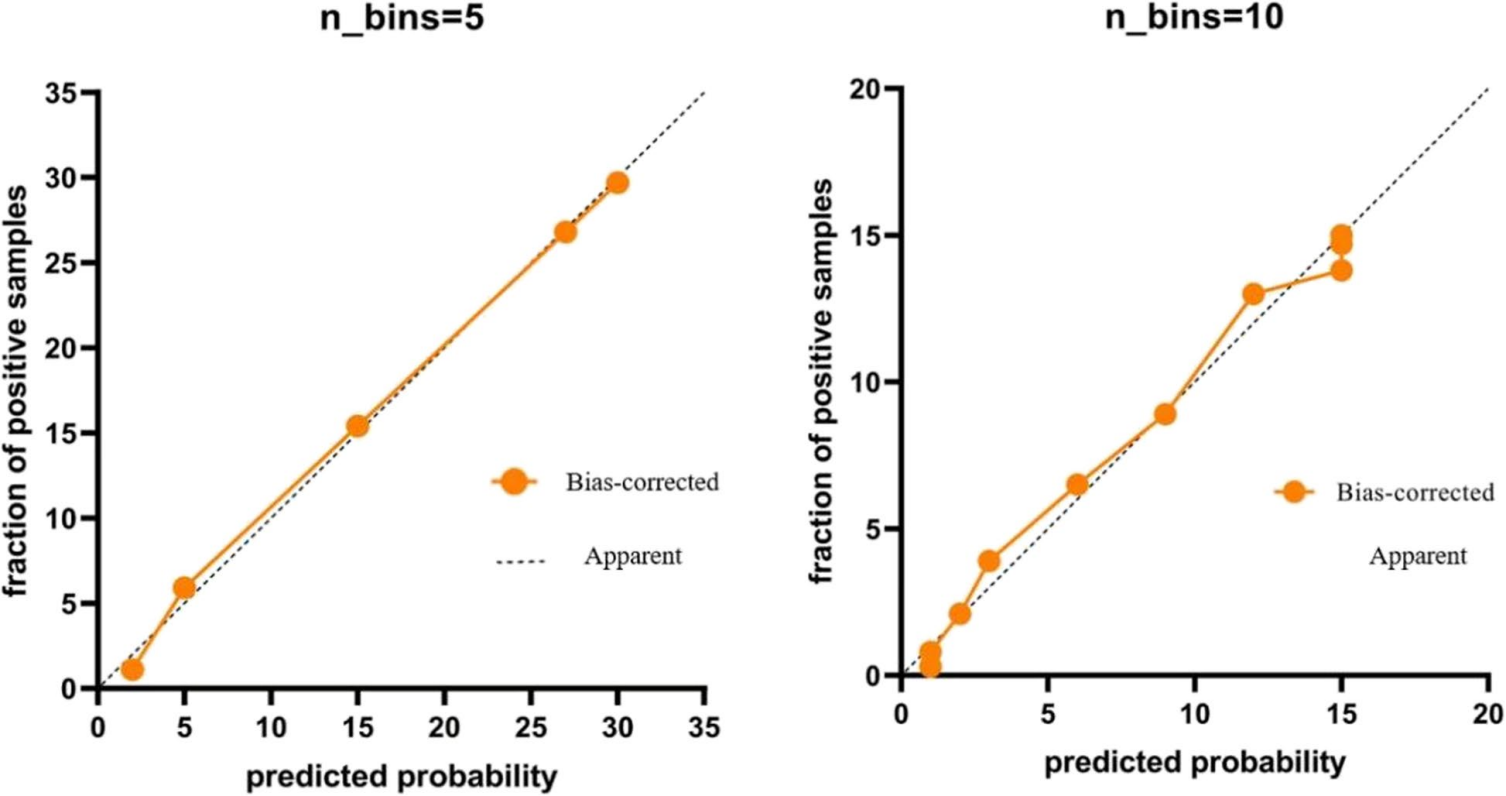
Calibration curves of nomogram

A prediction model was constructed based on the independent risk factors obtained by multivariate logistic regression analysis, and a nomogram was generated (Fig. 2). Based on the ROC curve of the prediction model (Fig. 3, Table 3), the sensitivity was 83.5%, the specificity was 93.2%, and the area under the ROC curve (AUC) of the combined predictor was 0.931, which was greater than that of any one of the independent risk factors (CRS-R score, DAO, PCT, ALB, and I-FABP). The data were divided into 5 groups for the Hosmer–Lemeshow (H–L) test (χ2 = 2.54, *P* = 0.468 > 0.05) and into 10 groups for the H–L test (χ2 = 9.98, *P* = 0.267 > 0.05). A calibration curve was drawn based on the test results, showing that the goodness-of-fit of the prediction model was good, and the prediction stability was good (Fig. 4).

**Table 3:** Area Under ROC curve

	AUC	Youden index	Sensitivity (%)	Specifity (%)	+ LR	-LR
Prediction model	0.931	1.767	83.5	93.2	12.279	0.177
CRS-R score	0.690	1.296	41.8	87.8	3.426	0.663
DAO	0.814	1.550	72.5	82.5	4.143	0.333
PCT	0.797	1.486	64.8	83.8	4.000	0.420
ALB	0.630	1.263	56.0	70.3	1.886	0.626
I-FABP	0.656	1.319	49.5	82.4	2.813	0.613

## Discussion

The gastrointestinal tract is an important digestive, endocrine and immune organ in the human body and is also the largest bacterial reservoir in the human body [7]. AGI secondary to pDOC, in addition to affecting the absorption and utilization of nutrients, will also lead to the translocation of toxins in the intestinal flora due to the destruction of the gastrointestinal mucosal barrier, aggravate the systemic immune inflammatory response, and eventually lead to septic shock and multiorgan failure [8]. Therefore, it is particularly important to predict the AGI of pDOC patients and intervene early. However, there is still a lack of clinical tools for the early prediction of secondary AGI in pDOC patients.

After exclusion according to the exclusion criteria, a total of 165 patients were included, and the results showed that AGI occurred in up to 55.15% of patients. According to previous research [9–11] and our clinical experience, this study selected 35 risk factors, covering many aspects, such as the degree of organ damage and treatment factors, in patients with chronic disorder of consciousness. Logistic regression analysis confirmed that CRS-R score, DAO, PCT, ALB, and I-FABP were risk factors for AGI in patients with pDOC.

The Brain Injury-Interdisciplinary Special Interest Group, Disorders of Consciousness Task Force [12] completed an evidence-based retrospective study of the behavioral scale for patients with consciousness disorders in 2010, and believed that the CRS-R was the most acceptable of all scales. The CRS-R is currently considered to be effective for assessing pDOC [13–15]. Therefore, in this study, CRS-R was used to evaluate the state of consciousness of patients.Research suggests that there is a bidirectional neuromodulation pathway between the brain and the gut, that is, the brain-gut axis (BGA). It is mediated by immune factors [16, 17]. When the brain is damaged by irreversible ischemia and hypoxia, the function of the cerebral cortex decreases, resulting in a decline in the ability of the central nervous system to regulate the enteric nervous system, and the function of the enteric nervous system is relatively independent. This increased sensitivity is more likely to cause gastrointestinal dysfunction. This study found that there is a significant correlation between the CRS-R score and AGI at admission, and multivariate logistic regression analysis showed that CRS-R score is one of the independent predictors of AGI in pDOC patients. Gastrointestinal dysfunction is likely to occur in these patients.

I-FABP is a group of low-molecular-weight proteins that are highly specifically distributed in mature small intestinal mucosal epithelial cells. Under normal circumstances, serum I-FABP levels are very low, but in early intestinal ischemia, epithelial cells are damaged. When the permeability increases (≤ 2 h), I-FABP can be released into the blood through the cell membrane surface and has high stability, so it is suitable as a marker of early damage to intestinal mucosal cells [18]. As an oxidase in the cytoplasm of villous epithelial cells, DAO is highly active. Its activity is closely related to the nucleic acid and protein synthesis of mucosal cells. It can reflect the integrity and repair ability of intestinal epithelial cells and is sensitive to the assessment of early intestinal mucosal damage [19]. Therefore, the increase in blood I-FABP and DAO levels is closely related to the occurrence of AGI. This study concluded that for every 1 μg/L increase in I-FABP, the possibility of AGI in patients with chronic disorder of consciousness increased by 0.223, and for every 1 mmol/L increase in DAO, the possibility of AGI in pDOC patients increased by 3.561 times, which deserves early clinical attention.

PCT is the calcitonin polypeptide precursor produced by thyroid C cells. Under pathological conditions, such as trauma, bacterial infection, and cardiogenic shock, organs and tissues other than the thyroid are stimulated to produce PCT in the blood, which can be detected within 2 h. It has high sensitivity and specificity and is a commonly used clinical indicator of inflammation. Patients with chronic disorder of consciousness often have low immune function. Once AGI occurs, intestinal ischemia and hypoxia, endotoxin release, and intestinal bacterial translocation lead to infection, all of which can lead to an increase in PCT. A study [20] showed that with the increase in AGI grade, the level of PCT increased significantly, and AGI may be a precipitating and stimulating factor of SIRS and MODS. This study found that there is a significant correlation between PCT and AGI, and multivariate logistic regression analysis showed that PCT is one of the independent predictors of AGI in patients with pDOC. For every 1 ng/mL increase in PCT, the possibility of AGI increases by 5.144 times.

Serum ALB levels are a common indicator reflecting the nutritional status of the body, and ALB helps to maintain intravascular osmotic pressure, promotes the transport of substances, and scavenges free radicals. ALB is reduced in acute inflammation, and hypoalbuminemia is associated with systemic inflammatory responses [11]. A systematic review of 4190 critically ill patients [21] found that reduced serum ALB levels were associated with all-cause mortality. Plasma colloid osmotic pressure decreases due to hypoalbuminemia, resulting in edema of tissues and organs. When mucosal edema occurs in the gastrointestinal tract, it easily causes acute gastrointestinal dysfunction.

Previous studies [22] have shown that logistic regression models fitting multiple indicators to establish a prediction model can significantly improve the predictive ability of the target outcome. Risk factors were used to establish a joint prediction model to predict the occurrence probability of AGI in pDOC patients. The ROC curve analysis showed that the AUC of the joint prediction model was 0.931, which was higher than the AUC of any single independent influencing factor, with a sensitivity of 83.5% and a specificity of 93.2%, which proved that the joint prediction model had high predictive value and was superior to any single predictor. The data were subjected to the H–L test, all *P* > 0.05, and a calibration curve was drawn based on the test results, indicating that the prediction model had good goodness-of-fit and good prediction stability.

In conclusion, the occurrence of AGI in pDOC patients involves many aspects, such as intestinal ischemia and hypoxia, immune response imbalance, and mucosal barrier damage, which is the result of the combined effect of multiple factors. The AGI prediction model for pDOC patients established in this study can be used in the clinic and helps to predict the occurrence of AGI in pDOC patients. For patients with potential risk of AGI, relevant intervention measures should be taken early, such as early enteral nutrition, post-pyloric feeding, and avoiding the use of drugs that impair gastrointestinal function.Nevertheless, this study also has certain limitations. First, as a single-center retrospective study, the sample size of this study was relatively small, and the results of the study may be affected by potential confounding factors and selection bias. Further research with multicenter and large samples is needed. For age-stratified studies, the predictive power of the prediction model in pDOC patients of different ages remains to be further validated.

## Data Availability

The datasets used and/or analyzed during the current study are available from the corresponding author on reasonable request.

## Abbreviations

pDOC: Prolonged disorder of consciousness
AGI: Acute gastrointestinal injury
ROC: Receiver operating characteristic
H–L: Hosmer–Lemeshow
VS: Vegetative state
UWS: Unresponsive wakefulness syndrome
MCS: Minimally conscious state
SIRS: Systemic inflammatory response syndrome
MODS: Multiple organ dysfunction syndrome
PCT: Procalcitonin
CRP: C-reactive protein
SAA: Serum amyloid A
DAO: Diamine oxidase
IFABP: Intestinal fatty acid binding protein
SOFA: Sequential Organ Failure Score
CRS-R: The Coma Recovery Scale–Revised
ALB: Albumin
WBC: White blood cell count
TBI: Traumatic brain injuries
